# Supplementary material for: Evaluation of the Abilities of Three Kinds of Copper-Based Nanoparticles to Control Kiwifruit Bacterial Canker
Source: Antibiotics (Basel). 2022 Jul 4;11(7):891. doi: 10.3390/antibiotics11070891 (PMC9312301; doi:10.3390/antibiotics11070891)
Supplement: Supplementary file 1 [file antibiotics-11-00891-s001.zip › antibiotics-1782104-supplementary.pdf]

Supplement materials

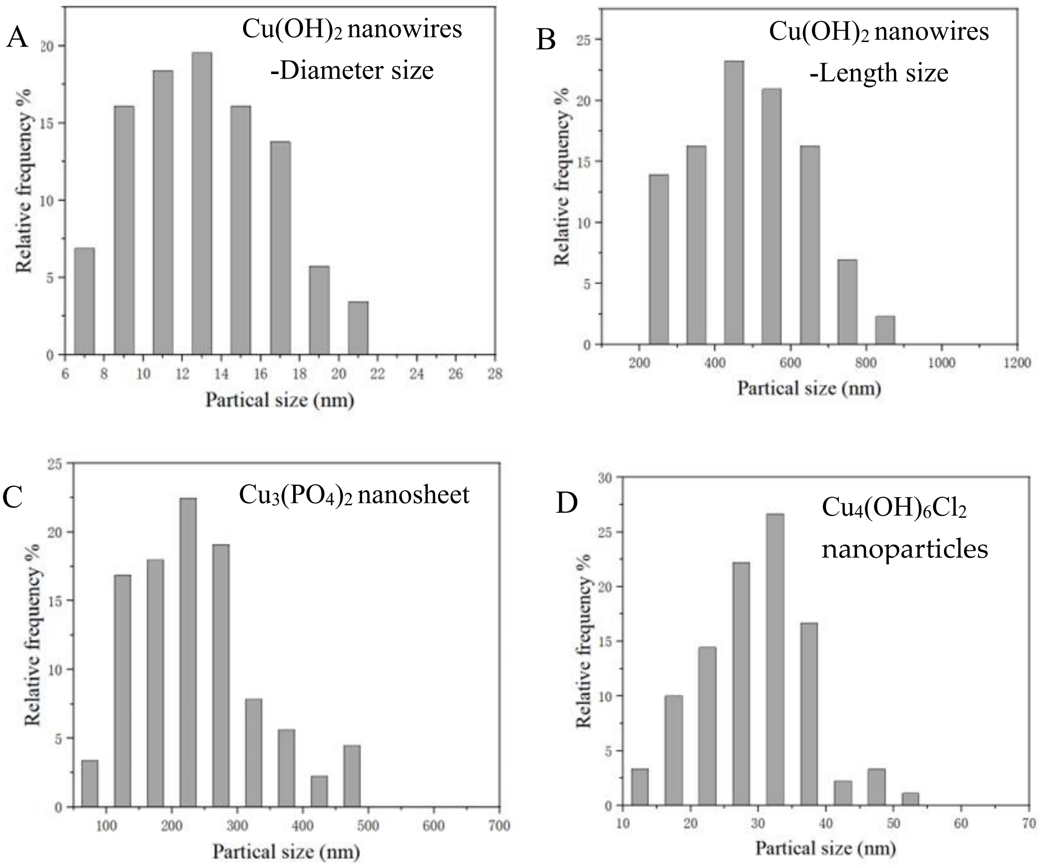

Figure S1. Size distribution of three kinds of copper-based nanoparticles. A. $Cu(OH)_2$ nanowires diameter statistical histogram; B. $Cu(OH)_2$ nanowires length statistical histogram; C. Histogram of length statistics of $Cu_3(PO_4)_2$ nanosheets; D. Histogram of $Cu_4(OH)_6Cl_2$ Nanoparticle Size Statistics.

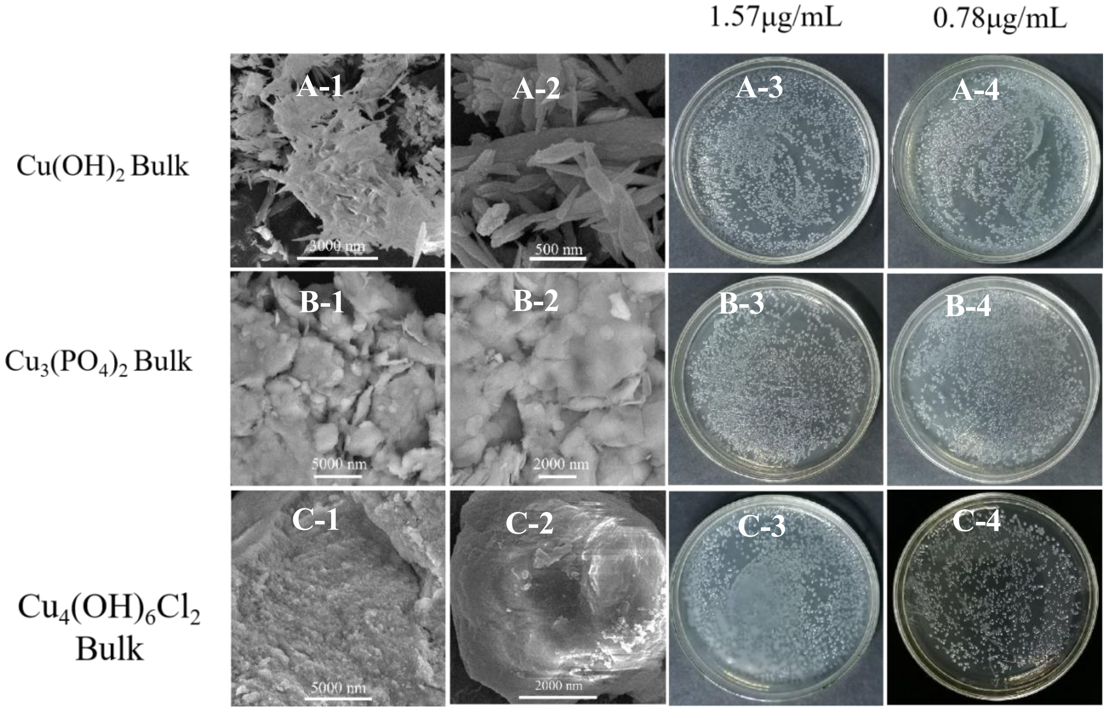

Figure S2. Characterization(A, B, C) of commercial copper-based compounds $Cu(OH)_2$, $Cu_3(PO_4)_2{\cdot}3H_2O$, and $Cu_4(OH)_6Cl_2$ and their inactivation efficiency($Cu(OH)_2$: A-3, A-4; $Cu_3(PO_4)_2{\cdot}3H_2O$: B-3, B-4, $Cu_4(OH)_6Cl_2$: C-3, C-4) of Psa.

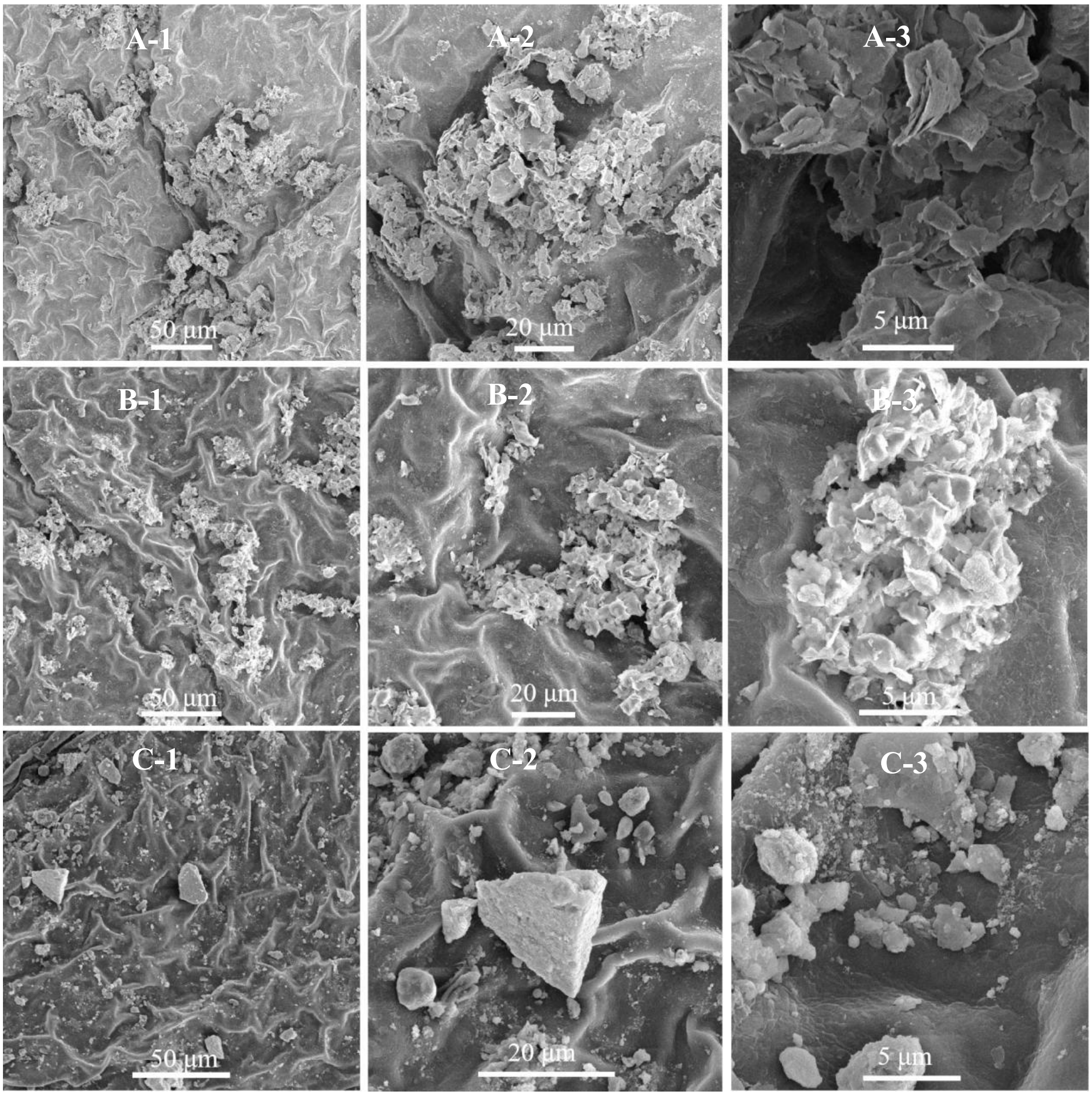

Figure S3. The distribution of three kinds of commercial copper-based compounds on the surface of kiwifruit leaves. A. Bulk $Cu(OH)_2$; B. Bulk $Cu_3(PO_4)_2$; C. Bulk $Cu_4(OH)_6Cl_2$.

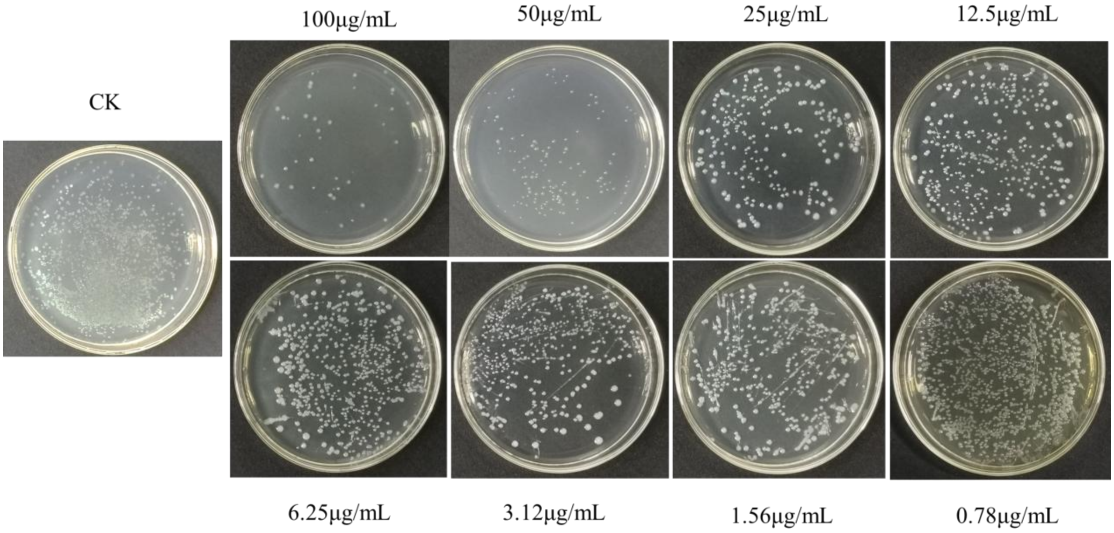

Figure S4. The MBC of the positive control agent thiodiazole copper.

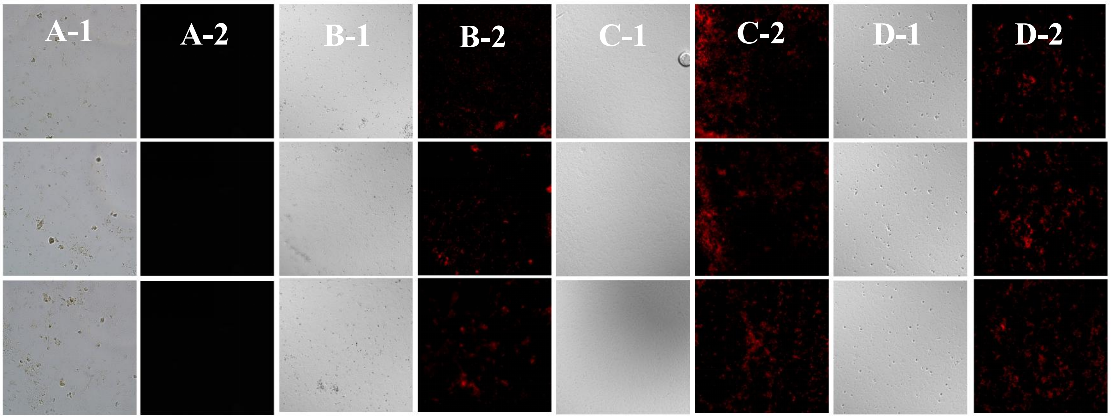

Figure S5. Supplementary fluorescence images for the membrane permeability test of Psa . A-1, B-1, C-1, D-1 bright field image; A-2, B-2, C-2, D-2 dark field image;Thiodiazole copper(A-1,A-2),$Cu(OH)_2$ nanowires (B-1, B-2), $Cu_3(PO_4)_2{\cdot}3H_2O$ nanosheets (C-1, D-2) and $Cu_4(OH)_6Cl_2$ nanoparticles (C-1, D-2).

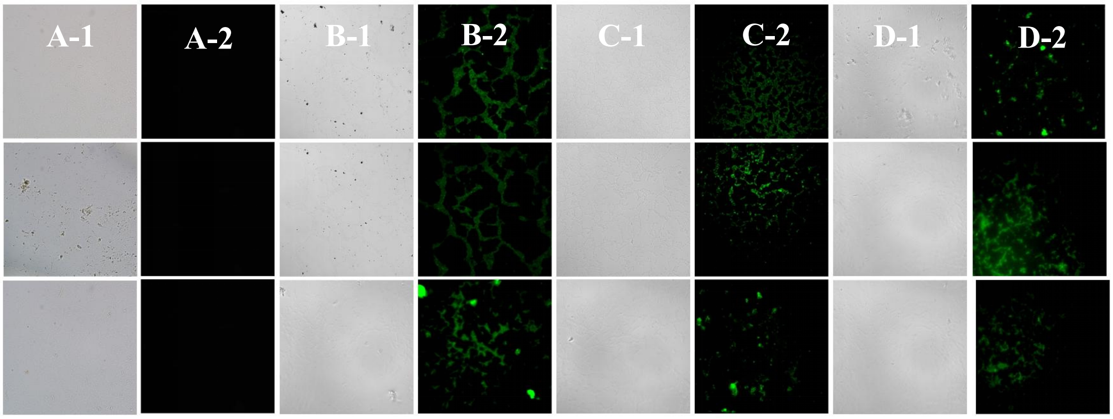

Figure S6. Supplementary fluorescence image for reactive oxygen species (ROS) accumulation test of Psa. A-1, B-1, C-1, D-1 bright field image; A-2, B-2, C-2, D-2 dark field image; Thiodiazole copper(A-1,A-2),$Cu(OH)_2$ nanowires (B-1, B-2), $Cu_3(PO_4)_2{\cdot}3H_2O$ nanosheets (C-1, D-2) and $Cu_4(OH)_6Cl_2$ nanoparticles (C-1, D-2).

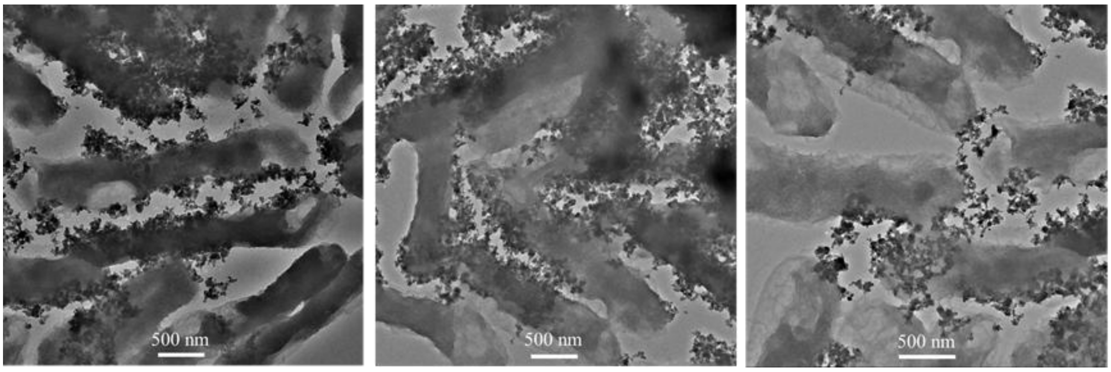

Figure S7. Supplementary TEM images for the interaction between Psa and $Cu_4(OH)_6Cl_2$ nanoparticles.

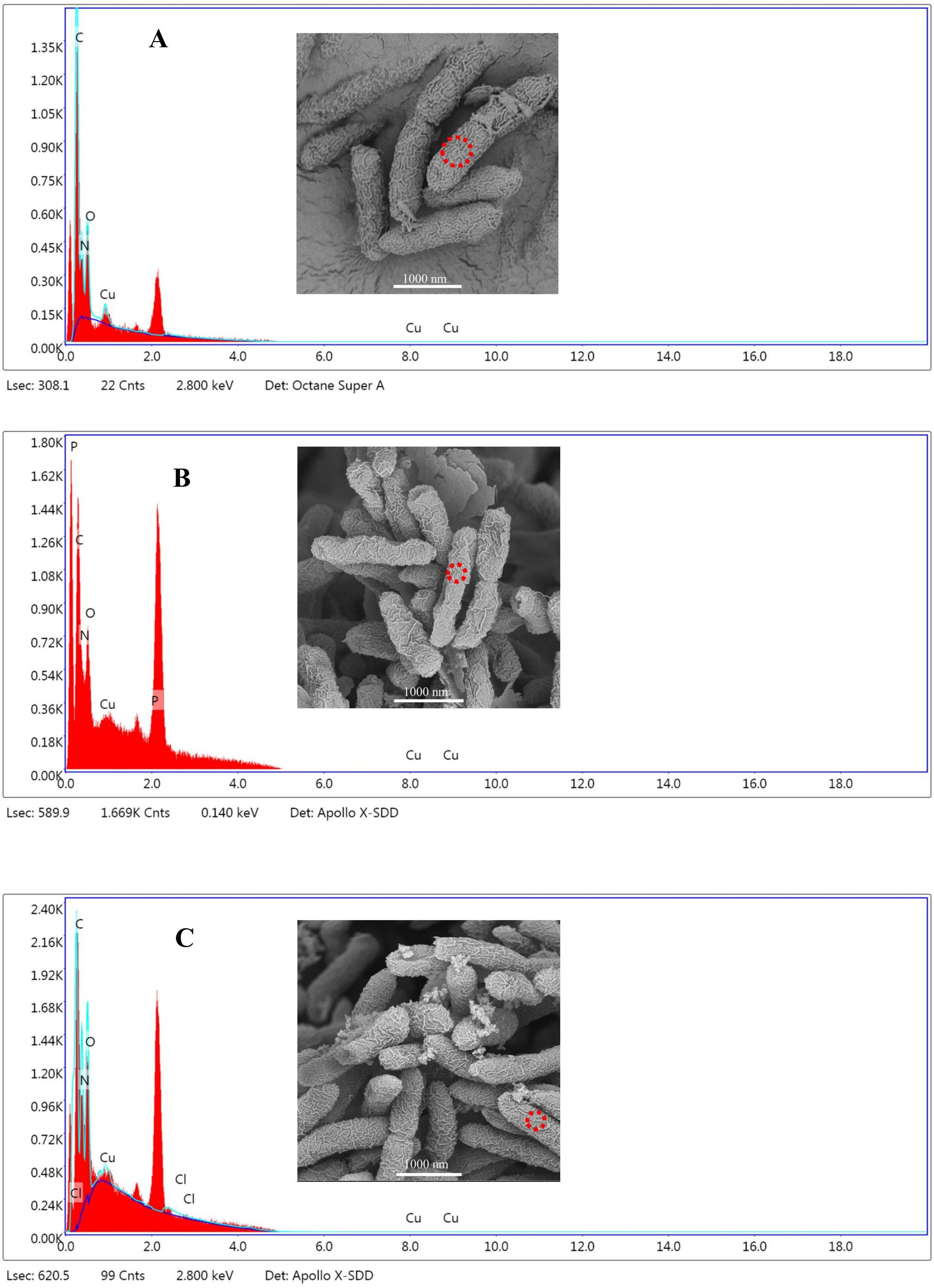

Figure S8. Supplementary SEM images and EDS spectrogram for the interaction between Psa and nanoparticles. A. $Cu(OH)_2$; B.$Cu_3(PO_4)_2$; C. $Cu_4(OH)_6Cl_2$.

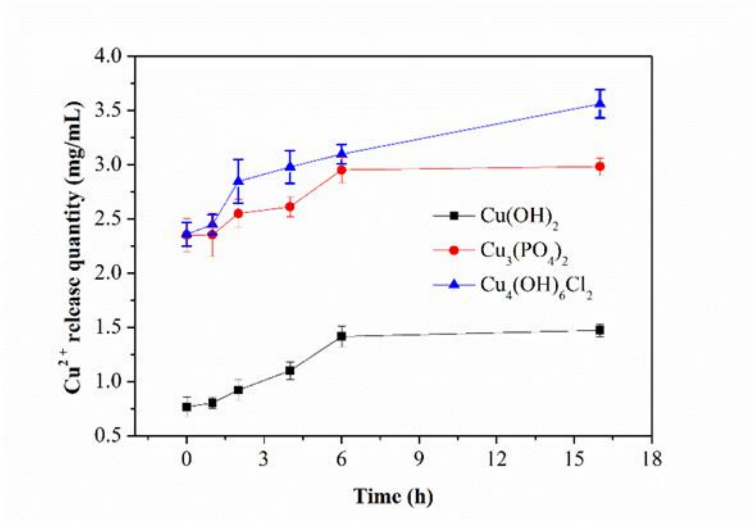

Figure S9. Concentration of Cu^{2+} in solution vs time after the indicated nanomaterials was introduced into aqueous solution at a mass concentration of 200 μg/mL.

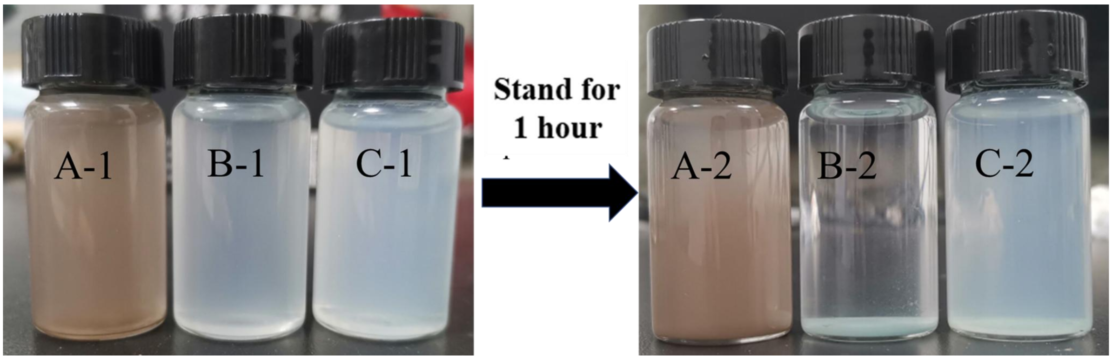

Figure S10. Colloidal stability test of copper-based nanoparticles dispersion. A. $Cu(OH)_2$; B.$Cu_3(PO_4)_2$; C. $Cu_4(OH)_6Cl_2$.

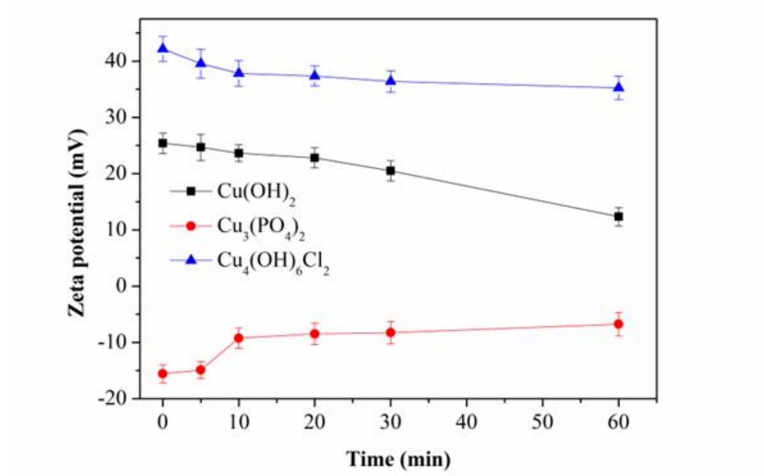

Figure S11. Zeta potential vs time of nanomaterials solution at a mass concentration of 200 μg/mL.
